# Life as an early-career researcher: interview with Amy Dawson

**DOI:** 10.4155/fsoa-2016-0052

**Published:** 2016-09-16

**Authors:** Amy Dawson

**Affiliations:** 1 VWR International Ltd, Hunter Boulevard, Magna Park, Lutterworth, LE17 4XN

**Keywords:** early career, IBD, inflammatory bowel disease, microfluidics, tissue culture

**Amy Dawson talks to Francesca Lake, Managing Editor.** Amy Dawson is a postdoctoral researcher using microfluidic technology to develop a dual-flow device for the maintenance of human intestinal tissue in a known orientation. The dual-flow maintains the two surfaces of the intestine separately with the aim focused on the treatment of inflammatory bowel disease (IBD) and establishing the interaction of the tissue with the commensal bacteria present in the gut. This is part of a collaborative study with Scarborough General Hospital and the Østfold Hospital Trust, Norway. Dr Dawson studied a BSc, MSc in biomedical science and a PhD in chemistry at the University of Hull, Hull, UK.

## Q Can you tell us a little about your career path to date?

I worked at the University of Hull for nearly 10 years. I started with a degree in biomedical science, followed by a masters in research in biological science looking at the presence of cardiac stem cells and if they differed through aging. I then moved into chemistry and studied a PhD in microfluidics. The project was based on making channels in a piece of filter paper that could then be used to measure iron levels of patients serum/plasma samples to detect anemia. This then led to my postdoctoral research associate position working in microfluidics and the maintenance of human intestinal tissue on a chip.

## Q What are you working on at the moment?

My current project is looking at IBD such as Crohn's disease or ulcerative colitis. Currently, there is no cure, only treatments that can maintain remission for prolonged periods of time; however, this is not applicable to everyone and sometimes the only treatment option available is major surgery to remove the affected parts of the intestines with the addition of a colostomy bag or stoma, which in some cases can be temporary, however in a lot it is permanent.

To look into IBD, we use as animal or cell culture models, which fail to fully replicate the disease we see in humans. My research is aimed at taking tissue from human patients undergoing a bowel resection – we then cut 5-mm biopsies to put into our dual-flow microfluidic system, which keeps the tissue in a known orientation (lumen surface to serosal surface) with continuous perfusion of nutrients into the tissue and simultaneous removal of waste products, mimicking the *in vivo* environment. This project and device has the final goal (once fully characterized) of being able to study various treatments – for example, can luminally applied bacteria alleviate the symptoms of IBD and could this be applied to a patient?

## Q What do you find most rewarding about your work?

The things I find most rewarding are being able to discuss with fellow researchers about my research and work at the University, either at group meetings or national and international conferences; exchanging ideas and working with both patients and surgeons to obtain samples and the day-to-day running of the laboratory.

## Q And what do you find the most challenging?

Maintaining a balance between teaching undergraduate students alongside project work, as tissue samples can come at any time of the day once the theater rings to say it is ready. Work has to be prioritized in order to achieve the end goals.

## Q Where do you see yourself in 20 years’ time?

In 20 years’ time (apart from having a house and a partner), I would like to have a permanent position either within academia or within industry – a job that has good scope for progression and is challenging.

## Q What advice would you give someone who is considering going into academia?

For someone going into academia, it is a hard slog and not to be taken lightly. I have spent the first 3 years since finishing my studies working on fixed-term contracts; the longest contract was 18 months. Throughout this, the aim has not only been to complete the set project but also to gain extra funding as the project progresses; gaining funding is very competitive and difficult.

It is good to have a supervisor who supports your aspirations, helps in grant writing/writing papers and supports you in attending conferences. At the same time, it is also important to maintain a good balance in work and life as it is so easy to spend evenings and weekends working and getting carried away.

## Q In your opinion, what would you say are the biggest challenges facing early-career researchers?

The biggest challenge is to gain funding for projects and work as to get a permanent job in such a competitive area is very hard; you have to have a proven track record of getting funding and having papers published while keeping up with demands in the lab, such as COSHH forms and ethics paperwork, which can easily fall behind or be forgotten about.

Not having a permanent job can also have drawbacks in others areas of life as well such as not been able to get a mortgage.

## Q What advice would you give to early-career researchers concerned about job stability?

Funding bodies such as the Wellcome Trust or NC3Rs which have fellowships aimed at young researchers, such as The David Sainsbury's Trust, now have a funding available for a fellowship specifically for a researcher who is within 3 or 5 years of finishing a PhD.

Applying for smaller funds as well as for equipment, for example, can help strengthen your CV, as can taking part in outreach events etc.

## Q If you were given unlimited funding, what would you do with it?

If I was given unlimited funding, I would have my own research lab and research group including PhDs, postdocs, technicians and undergraduate students working within IBD. The lab would be top of the range and includes all containment levels required for cell work, tissue work and bacterial work. There would be a direct link between the Hospital and the lab with scope for monthly meetings in the group and ability to travel to all conferences of relevance. Staff and students would have office space, either shared or individual depending on level, to write and do research. Research would not just focus on maintaining tissue on a chip but all aspects of IBD including the microbiome, food intake and how our diet can have an effect and treatment options for IBD patients.

